# Monitoring the heme iron state in horseradish peroxidase to detect ultratrace amounts of hydrogen peroxide in alcohols†

**DOI:** 10.1039/d1ra00733e

**Published:** 2021-03-09

**Authors:** Raheleh Ravanfar, Alireza Abbaspourrad

**Affiliations:** Department of Food Science, Cornell University Ithaca NY USA alireza@cornell.edu

## Abstract

Despite the importance of hydrogen peroxide (H_2_O_2_) in initiating oxidative damage and its connection to various diseases, the detection of low concentrations of H_2_O_2_ (<10 μM) is still limited using current methods, particularly in non-aqueous systems. One of the most common methods is based on examining the color change of a reducing substrate upon oxidation using UV/Vis spectrophotometry, fluorophotometry and/or paper test strips. In this study, we show that this method encounters low efficiency and sensitivity for detection of ultratrace amounts of H_2_O_2_ in non-aqueous media. Thus, we have developed a simple, fast, accurate and inexpensive method based on UV/Vis spectrophotometry to detect H_2_O_2_ in non-aqueous systems, such as alcohols. In this regard, we demonstrate that monitoring the Soret and Q-band regions of high-valent iron-oxo (ferryl heme) intermediates in horseradish peroxidase (HRP) is well suited to detect ultratrace amounts of H_2_O_2_ impurities in alcohols in the range of 0.001–1000 μM using UV/Vis spectrophotometry. We monitor the optical spectra of HRP solution for the red shift in the Soret and Q-band regions upon the addition of alcohols with H_2_O_2_ impurity. We also monitor the reversibility of this shift to the original wavelength over time to check the spontaneous decay of ferryl intermediates to the ferric state. Thus, we have found that the ferryl intermediates of HRP can be used for the detection of H_2_O_2_ in alcohols at μg L^−1^ levels through *via* UV/Vis spectrophotometric method.

## Abstract 2

Despite the importance of hydrogen peroxide (H_2_O_2_) in initiating oxidative damage and its connection to various diseases, the detection of low concentrations of H_2_O_2_ (<10 μM) is still limited using current methods, particularly in non-aqueous systems.

## Graphical abstract

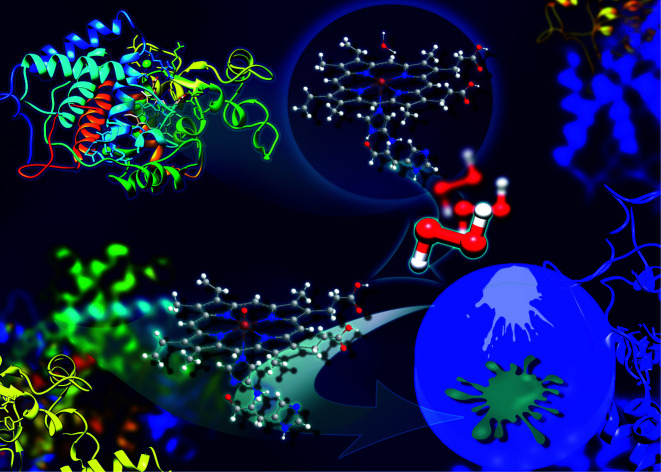

## Introduction

Hydrogen peroxide (H_2_O_2_) is an essential oxygen metabolite in living systems and serves as a messenger in cellular signal transduction.^1^ The overproduction of H_2_O_2_ from the mitochondrial electron transport chain results in oxidative stress, causing functional decline in organ systems.^2^ Such oxidative stress over time is also connected to various diseases, including cancer,^3^ cardiovascular disorders,^4^ and Alzheimer's disease and related neurodegenerative diseases.^5^ Moreover, H_2_O_2_ and its derivatives are strong oxidizing agents employed in many industrial and medical processes, such as the synthesis of organic compounds and disinfection.^6,7^ The significant impact of H_2_O_2_ on a variety of oxidative damage mechanisms,^8,9^ environmental hazards,^7,10^ and human health,^11,12^ as well as its application in biosensing,^13^ provide motivation to develop a sensitive and selective diagnostic method for detecting and quantifying H_2_O_2_, particularly at low concentrations.

Over the past several decades, many H_2_O_2_ sensing techniques have been devised based on spectrophotometry,^14,15^ fluorescence,^12,16^ chemiluminescence,^17^ enzymatic, and electrochemical methods.^18–22^ One of the most extensively used enzymatic systems for H_2_O_2_ sensing is horseradish peroxidase-H_2_O_2_ system (HRP-H_2_O_2_).^21–26^ The broad application of horseradish peroxidase (HRP) in H_2_O_2_ sensing is due to its ability to translate catalysis into an electrochemical signal, as well as its stability and commercial availability.^27^ HRP is able to catalyze the heterolytic cleavage of the peroxidic bond in H_2_O_2_ and form a high-valent iron-oxo (ferryl heme) intermediate of the enzyme (compound I).^28–31^ In compound I, the iron at the heme center has been oxidized from Fe^III^ to Fe^IV^=O, and the porphyrin or an amino acid in the side chain of HRP is oxidized to a radical.^32^ Thus, compound I can oxidize two molecules of a reducing substrate, such as ABTS (2,2′-azino-bis(3-ethylbenzothiazoline-6-sulfonic acid)), through two consecutive single electron reactions to form compound II, before finally being reduced back to the Fe^III^ state.^32–39^

Most HRP-based methods, however, are limited by some serious disadvantages, such as environmental instability, complex fabrication design, tedious immobilization procedures, and high cost.^15,18^ For example, 3,3′,5,5′-tetramethylbenzidine/HRP-based method to detect H_2_O_2_ is mainly based on the use of 3,3′,5,5′-tetramethylbenzidine (TMB), which is the most commonly used chromogen for HRP. TMB performs as a reducing organic substrate and is oxidized by ferryl intermediates formed upon the reaction of HRP and the H_2_O_2_ impurity in the media.^40^ Moreover, most of these methods are only efficient for H_2_O_2_ detection in aqueous media and encounter low efficiency and sensitivity in organic solvents, such as alcohols. As previously reported, the partial oxidation of primary or secondary alcohols due to autoxidation results in the production of H_2_O_2_, which produces an aldehyde or ketone as a coproduct.^41^ Since alcohols are commonly used in various chemical and enzymatic reactions, the presence of unreported amounts of H_2_O_2_ can interfere with reaction cascades.^42^ Therefore, providing a selective, rapid, convenient, and low cost analytical method for detection of H_2_O_2_ in alcohols is of great interest.

Here, we report a simple spectrophotometric method to detect H_2_O_2_ in alcohols at μg L^−1^ levels through the direct detection of the ferryl intermediate of HRP. In this manner, we are able to detect ultratrace amounts of H_2_O_2_ in alcohols, such as ethanol, glycerol, 2-chloroethanol, and isopropanol. In this method, we monitor the red shift in the Soret and Q-band regions of the HRP's optical spectrum upon the addition of alcohols with H_2_O_2_ impurity to the HRP aqueous solution at pH 6.0. The red shift of the Soret band from 402 nm to 418 nm, and the appearance of two Q-bands at 527 nm and 557 nm are indicative of the formation of the ferryl intermediates of HRP, which can be formed only in the presence of H_2_O_2_ impurity.^43–45^ Thus, we consider the change in the Soret band of Fe(iii) and formation of Fe(iv) ferryl intermediates as confirmation of the presence of H_2_O_2_ impurity. Furthermore, we monitor the reversibility of the red shifts over time to their original wavelengths as an indication of the spontaneous decay of the ferryl intermediates to the ferric state, distinguishing these red shifts from possible solvatochromic shifts. Our method is based on the use of HRP by itself, and does not need any reducing substrate such as ABTS and TMB. We monitor the formation of ferryl intermediates upon the reaction of HRP and H_2_O_2_ impurity in the media. Using this method, we can efficiently detect μg L^−1^ levels of H_2_O_2_ (0.001–1000 μM) in alcohols, where it is barely possible to detect this amount of H_2_O_2_ using other common methods, such as hydrogen peroxide test strips. We characterize the ferryl intermediates and their decay to ferric heme upon the addition of alcohols to HRP using UV/Vis spectrophotometry and confirm their presence using electron paramagnetic resonance (EPR) and cyclic voltammetry (CV). This demonstration suggests the importance of monitoring the ferryl intermediates for the detection of H_2_O_2_ impurity in alcohols at μg L^−1^ levels using a simple, cost-effective, and accurate method.

## Results and discussion

**Fig. 1:**
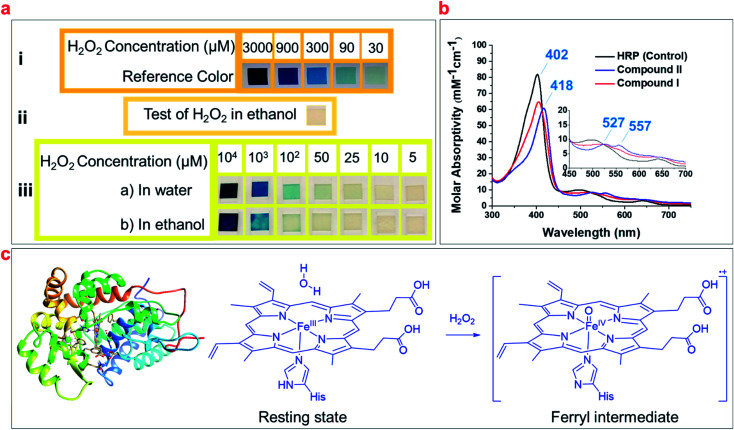
(a) The detection and quantification of H_2_O_2_ according to the reference colors using H_2_O_2_ test strips: (i) reference colors with a detection limit of 30 μM; (ii) examination of ethanol for H_2_O_2_ impurity using the test strip; (iii) comparison of the detection limit of H_2_O_2_ in ethanol and water using the test strips. (b) The UV/Vis spectra of the resting state of HRP (control), in comparison with its ferryl intermediates (compound I and compound II). (c) The schematic of HRP (PDB code: 1ATJ), and its heme structure in the resting state and ferryl intermediate upon reacting with H_2_O_2_.

Common hydrogen peroxide test strips were used to detect H_2_O_2_ impurity in a 100% ethanol sample, the minimum detectability of these strips is reported as 30 μM H_2_O_2_ (Fig. 1a(i)). Fig. 1a(ii) illustrates that the strips showed no detectable hydrogen peroxide in ethanol. We prepared different concentrations of H_2_O_2_ in both water and pure ethanol, and compared the color of hydrogen peroxide test strips after exposure to these solutions (Fig. 1a, iii). The results showed the test strips barely detected H_2_O_2_ in ethanol at low concentrations (<100 μM), and cannot accurately quantify the concentrations of H_2_O_2_ (<1 mM) (Fig. 1a, iii). Thus, we aim to develop an accurate method to detect and quantify the H_2_O_2_ impurity in alcohols.

Studies have shown that the optical spectra of the resting state of HRP (Fe^III^) has a Soret band of 402 nm and a Q-band of 497 nm, while the ferryl heme intermediate of HRP (Fe^IV^=O) has a Soret of 418 nm and two Q-bands of 527 nm and 557 nm (Fig. 1b).^43–45^ We hypothesized that these indicative peaks could be used to detect the H_2_O_2_ impurity of alcohols and developed a simple spectrophotometric method utilizing HRP solutions to detect and quantify H_2_O_2_ based on the formation of ferryl intermediates (Fig. 1c).^44^

**Fig. 2:**
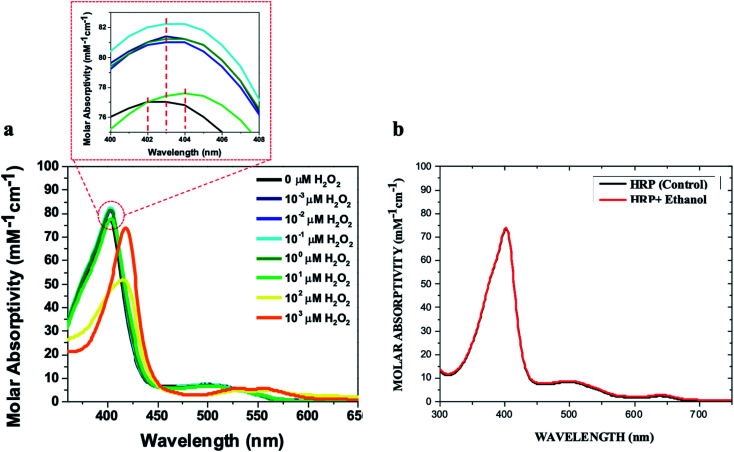
(a) UV/Vis spectra of the 10 μM HRP solution in 0.1 M phosphate buffer (500 μL) upon the addition of 500 μL of ethanol containing H_2_O_2_ at these concentrations (0 μM, 0.001 μM, 0.01 μM, 0.1 μM, 1 μM, 10 μM, 100 μM, and 1000 μM). (b) The UV/Vis spectra of the 10 μM HRP solution (500 μL) upon the addition of 500 μL of pure (H_2_O_2_-free) ethanol in comparison with control HRP solutions. All HRP solutions were prepared in 0.1 M potassium phosphate buffer (pH = 6.0).

We added different concentrations of H_2_O_2_ in ethanol to aqueous solutions of HRP at concentrations of 1 μM and 10 μM, and monitored the red shifts of the Soret and Q-bands relevant to the formation of ferryl intermediates. The results showed that 1 μM HRP solutions can be used to detect lower concentrations of H_2_O_2_ impurity in ethanol (0.001 μM) by making a red shift in the Soret band from 402 nm to 404 nm (Fig. S1a and b†), while a 10 μM HRP solution can be used to detect higher concentration of H_2_O_2_ impurity of ethanol (10 μM) by making a red shift in the Soret band from 402 nm to 404 nm (Fig. 2a and S2a–f†). The 1 μM and 10 μM HRP solutions exhibited a red shift from 402 nm to 418 nm upon the addition of 100 μM and 1000 μM H_2_O_2_ in ethanol, respectively (Fig. S1a–g and S2a–h†). Both concentrations of HRP solutions show Soret bands red shifted to 418 nm at higher concentrations of H_2_O_2_ in ethanol (Fig. S1h, i, S2i† and 2a), and only the 1 μM HRP solution is able to detect low concentrations of H_2_O_2_ impurity of ethanol (*e.g.* 0.001 μM). Thus, we suggest using the 1 μM HRP solution for detection of high and low H_2_O_2_ impurity in ethanol in the range of 0.001–1000 μM. The lower concentration of HRP is better for lower concentrations of hydrogen peroxide because less HRP is in resting ferric state.

**Fig. 3:**
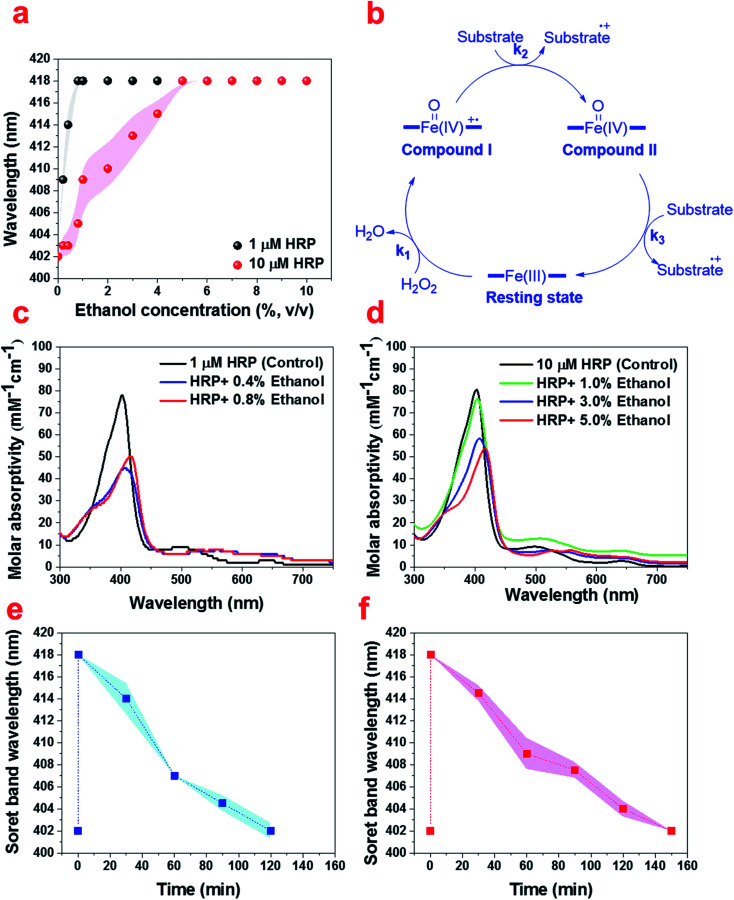
(a) The change in the wavelength of the Soret band through the titration of 500 μL of the HRP solution (1 μM and 10 μM) with varying amounts of ethanol known to contain 80 μM H_2_O_2_. (b) The schematic for the decay of ferryl intermediates to the ferric state. (c) Titration of 500 μL the HRP solution (1 μM) with ethanol known to contain 80 μM H_2_O_2_ to achieve a red shift of 418 nm. (d) Titration of 500 μL the HRP solution (10 μM) with ethanol known to contain 80 μM H_2_O_2_ to achieve 418 nm. (e) The reversibility of the red shift from 418 nm to 402 nm over 2 h for the HRP solutions treated with 10% (v/v) ethanol containing 80 μM H_2_O_2_. (f) The reversibility of the red shift from 418 nm to 402 nm over 2.5 h for 500 μL of the HRP solution treated with 20% (v/v) ethanol known to contain 80 μM H_2_O_2_.

The red shifts correlated to the ferryl intermediates can be distinguished from solvatochromic shifts. First, it is noteworthy that the addition of higher concentrations of ethanol containing H_2_O_2_ impurity do not increase the red shift to more than 418 nm (Fig. 3a). Second, the ferryl intermediates of HRP are not thermodynamically stable and decay spontaneously (Fig. 3b).^32,46^ Thus, the red shifts of the ferryl intermediates would be reversible. These two characteristics of the red shifts, correlated to the ferryl intermediates, can distinguish them from solvatochromic shifts. Even at lower concentrations of H_2_O_2_ in ethanol, the red shift can reach 418 nm upon the titration of the HRP solution (either 1 μM or 10 μM) with more ethanol, which adds more H_2_O_2_ to the system (Fig. 3c and d). However, over time (∼2 h) the Soret band moved back to 402 nm, showing that the ferryl intermediates decay to the iron(iii) state. Treating HRP solutions with different quantities of ethanol, all of which contain the 80 μM H_2_O_2_ impurity, we found that the time for the ferryl intermediates of HRP to decay to 402 nm increases from 2 h for 10% v/v ethanol to 2.5 h for 20% v/v ethanol in the reaction mixture (Fig. 3e and f). This increase in the decay time is because of the addition of more H_2_O_2_ in the reaction mixture along with the ethanol, which makes it possible for each molecule of HRP to produce ferryl intermediates for a longer time. Thus, the ferryl intermediates are accumulating in the reaction mixture for a longer period of time, and subsequently, the shifts take longer to return to their original wavelength.

To investigate the utility of this method beyond ethanol, we explored the detection of H_2_O_2_ impurity in several different alcohols. The UV/Vis spectra show that the Soret band of HRP red shifts to 418 nm upon the titration of the HRP solution with ethanol, glycerol, isopropanol, and 2-chloroethan-1-ol, with a shoulder at 350 nm, as well as Q-bands at 527 nm and 557 nm (Fig. S3a–d†). However, methanol, 2,2-dichloroethan-1-ol, 2,2,2-trichloroethan-1-ol, 2,2,2-trifluoroethan-1-ol, 2-mercaptoethan-1-ol, or ethylene glycol do not show any red shift in the Soret band or Q-band of HRP (Fig. S4†). This suggests that they do not contain any detectable H_2_O_2_ impurity. Interestingly, when ferryl intermediates decay to the ferric state over time, HRP can show the same red shift from 402 nm to 418 nm upon the second addition of these alcohols (Fig. S5a and b†). Thus, the HRP solution can be used as a recyclable method to detect H_2_O_2_ impurities. Moreover, the circular dichroism data shows that HRP conformation does not change at all during the ethanol treatment at both 1 μM and 10 μM HRP solution concentrations (Fig. S5c and d†).

**Fig. 4:**
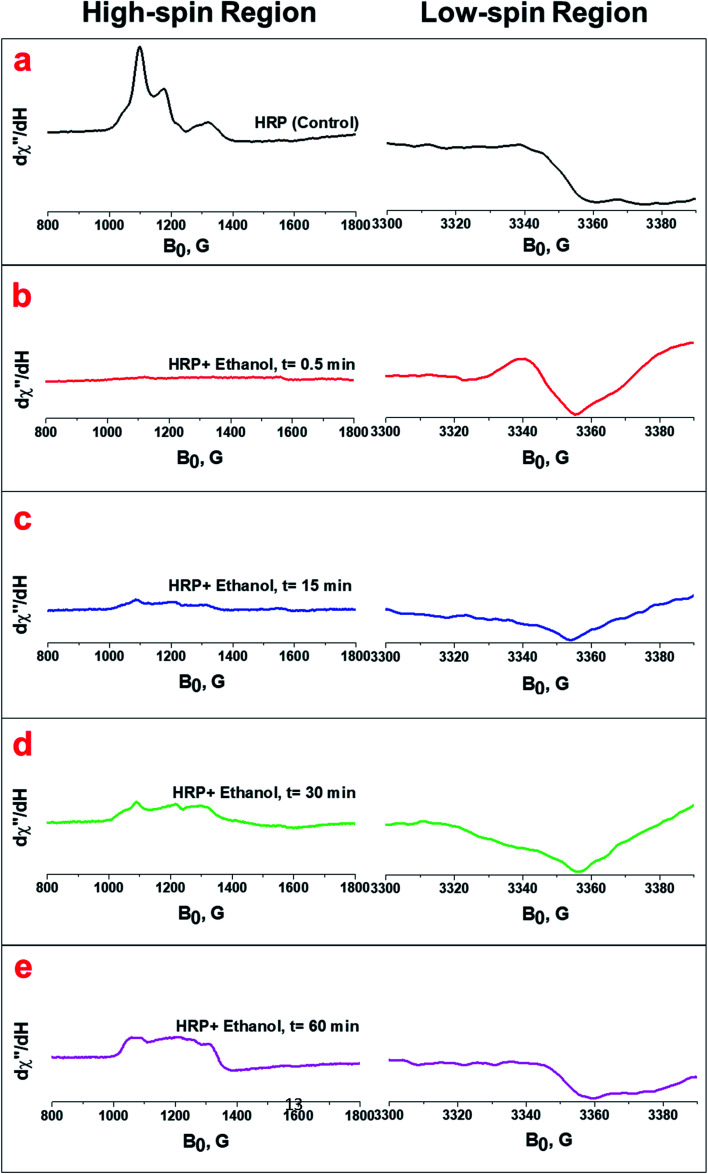
(a) EPR spectra of the native HRP (control) at both high-spin and low-spin regions. (b, c, d and e) EPR spectra of the HRP upon the addition of ethanol containing H_2_O_2_ impurity at 0.5 min, 15 min, 30 min, and 60 min, respectively. The spectra were collected at 12 K and 625 μW. The HRP solution (100 μL) was treated with 100 μL ethanol containing 80 μM H_2_O_2_.

Previous studies with HRP and H_2_O_2_ have shown that the ferryl intermediates of HRP formed through the reaction of ferric state of HRP with H_2_O_2_ are EPR-silent and generate only a broad EPR signal characteristic of the oxyferryl porphyrin π-cation radical (*g* = 2.0).^38,44,47,48^ EPR spectra for HRP solutions treated with ethanol (Fig. 4) and glycerol (Fig. S6†) containing H_2_O_2_ impurity confirm the formation of ferryl intermediates, which are also shown in the UV/Vis spectra shown earlier (Fig. S3a and b†). The HRP solution with pure ethanol does not show any formation of ferryl intermediates, based on the results from UV/Vis spectrophotometry (Fig. 2b). The initial formation of an EPR-silent intermediate upon the reaction of ferric HRP with alcohol molecules over a short timescale (30 s) shows that the ferric signals in native HRP (control) (Fig. 4a and S5a†) disappear in the high-spin region of heme while a new resonance is observed in the region of the low spin heme (*g* = 2.0) arising from a radical cation located on the heme (Fig. 4b and S5b†). The EPR spectra of the intermediates formed upon the addition of impure ethanol and glycerol to HRP solutions are also in agreement with previous reports of EPR analysis of ferryl intermediates upon the addition of H_2_O_2_ to HRP, ascorbate peroxidase, and cytochrome C peroxidase.^44,47,48^ Examining the EPR spectra for one hour, we found that the features of the high spin ferric heme (Fe^III^) begin to reappear over time, while the organic radical signal decays in the low-spin region (Fig. 4c–e, and S6c–e†). Thus, we also confirmed the decay of the ferryl intermediates through the reversibility of the EPR signals at both high-spin and low-spin regions.

**Fig. 5:**
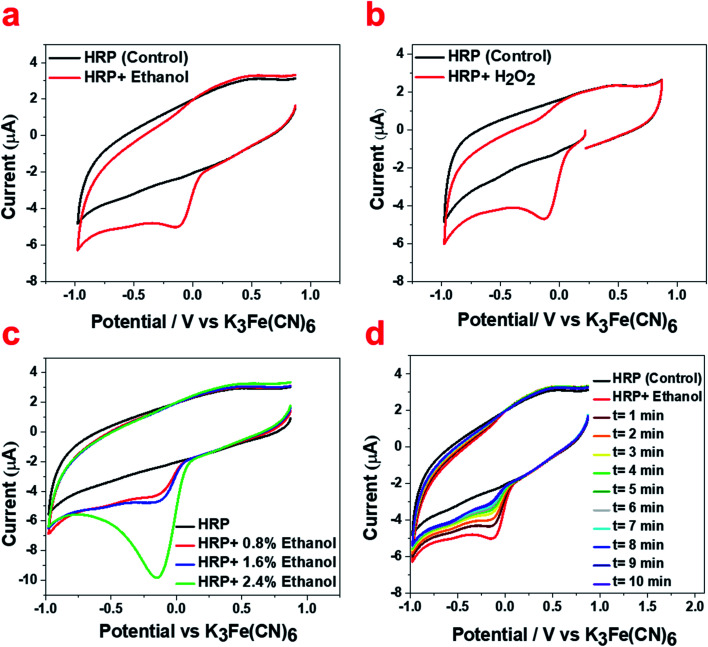
Electroreduction catalysis by HRP (control, black) upon treatment (red) with (a) ethanol known to contain 80 μM H_2_O_2_ and (b) aqueous solution of H_2_O_2_ (80 μM). (c) The increase of intensity of the reduction peak upon the addition of more ethanol percentage (v/v) containing 80 μM H_2_O_2_ impurity. (d) The decrease of the intensity of the reduction peak over a 10 min period for HRP treated with ethanol containing the known 80 μM H_2_O_2_ impurity. Cyclic voltammograms were measured in N_2_-saturated potassium phosphate buffer (0.1 M, pH 6.0) at a scan rate of 100 mV s^−1^.

We also confirmed the formation of the ferryl intermediate (Fe^IV^=O) and its decay to Fe^III^ using cyclic voltammetry. The cyclic voltammogram of the HRP solution shows the appearance of a reduction peak associated with the ferryl intermediate upon the addition of ethanol known to contain 80 μM H_2_O_2_ (Fig. 5a), which is the same as the reduction peak that appeared upon the addition of H_2_O_2_ aqueous solution to HRP solutions (Fig. 5b). The intensity of this reduction peak increases upon the addition of more ethanol percentage (v/v) containing the known 80 μM H_2_O_2_ impurity (Fig. 5c). The decay of the ferryl intermediate over time is also shown by the decreasing peak intensity over 10 minutes (Fig. 5d). This result is in agreement with the decay of the Fe^IV^=O intermediate demonstrated in the UV/Vis spectra (Fig. 3e–f) and EPR spectra (Fig. 4a–e), and the formation of ferryl intermediates upon the reaction of heme and H_2_O_2_.^46^

**Fig. 6:**
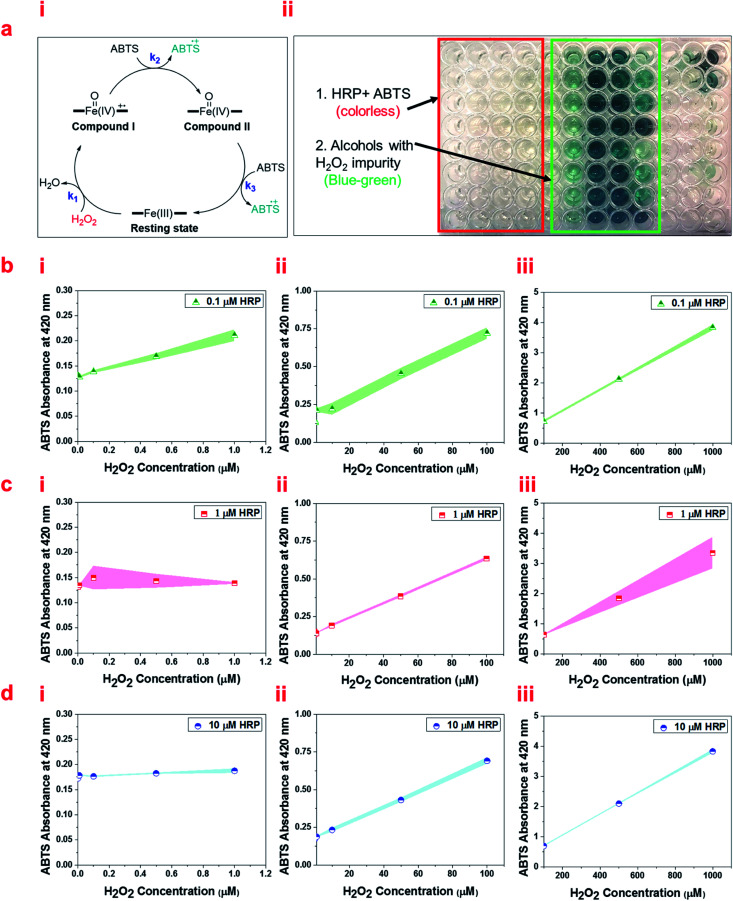
(a, i) The schematic of the oxidation of ferric heme to ferryl intermediates and subsequent reduction to ferric heme using electron transfer to oxidize ABTS. (ii) A 96-well plate containing HRP solutions and ABTS solutions upon the addition of alcohols with different concentrations of H_2_O_2_. (b) UV/Vis absorbance at 420 nm of 0.1 μM HRP, (c) 1 μM HRP solution, and (d) 10 μM HRP solutions reacted with 20 mM ABTS at different ranges of H_2_O_2_ impurity of ethanol (i) 0–1 μM, (ii) 1–100 μM, and (iii) 100–1000 μM. Briefly, 50 μL of ABTS in 0.1 M potassium phosphate buffer (pH 6.0) was mixed with 50 μL of HRP solution and 50 μL of ethanol containing different concentrations of H_2_O_2_. The absorbance at 420 nm (*λ*_max_ of the oxidized product of ABTS) *versus* H_2_O_2_ concentration was recorded at 25 °C 30 min after mixing, which was increased linearly.

We also quantify the detected H_2_O_2_ impurity in alcohols using ABTS as a reducing organic substrate in the aqueous solution of HRP (pH 6.0). ABTS can donate electrons to ferryl intermediates of HRP (Fig. 6a, i). ABTS, which has been widely used in the literature to measure HRP activity, changes from colorless to blue-green in color upon oxidation, and the intensity of this color can be easily measured by UV/Vis spectrophotometry (Fig. 6a, ii). Measuring the color intensity of ABTS (2 mM and 20 mM) 30 minutes after the reaction with HRP solutions (0.1 μM, 1 μM, and 10 μM) and ethanol containing different concentrations of H_2_O_2_ impurity, we optimized the best concentrations for measuring each range of H_2_O_2_ impurity. Our results show different trends at different ranges of H_2_O_2_ impurity based on the HRP and ABTS concentrations. Using both 2 mM and 20 mM ABTS solutions, all HRP solution concentrations result in an increasing linear trend for the absorption of ABTS in the range of 1–1000 μM H_2_O_2_ impurity in ethanol (Fig. 6b–d and S7a–c†). However, 1 μM and 10 μM HRP solutions do not show a significant increasing trend in the range of 0–1 μM H_2_O_2_ impurity in ethanol (Fig. 6c, i and d, i, and S7b, i and c, i†). Fig. 6b, illustrates that at 20 mM ABTS solution, 0.1 μM HRP solution shows a significant increase in absorption along with the increase in concentration of the H_2_O_2_ impurity in ethanol in the range of 0–1 μM. The 2 mM ABTS, however, is not appropriate for measuring the H_2_O_2_ impurity of ethanol in the range of 0–1 μM due to the non-significant change of absorption along with the concentration of the H_2_O_2_ impurity of ethanol (Fig. S7a, i†). Thus, we suggest using 0.1 μM HRP in a solution of 20 mM ABTS to quantify the H_2_O_2_ impurity in alcohols in the ranges of 0–1000 μM.

Since the slope of the graphs for ABTS absorption *versus* H_2_O_2_ concentration in the range of 0–1000 μM follows a step gradient, we propose three formulas to measure the accurate level of H_2_O_2_ impurity in alcohols in the range of 0–1 μM, 1–100 μM, and 100–1000 μM, individually (Table S1†). These formulas are derived from the linear fits based on the UV/Vis absorbance of ABTS at 420 nm for three ranges: 0–0.3 a. u., 0.3–1 a. u., and 1–4 a. u. Using these formulas based on the absorbance of ABTS, we successfully measured the H_2_O_2_ impurity in a few common primary and secondary alcohols (Table S2†).

In conclusion, we demonstrate that the ferryl intermediates of HRP can be used for the detection of H_2_O_2_ in alcohols at μg L^−1^ levels using UV/Vis spectrophotometry. The red shift in the Soret band in the optical spectra of the HRP solution from 402 nm up to 418 nm upon the addition of alcohols was measured and it was shown to be reversible over time. Using this method, we can efficiently detect μg L^−1^ levels of H_2_O_2_ impurity in alcohols, where it is barely possible using other common methods such as hydrogen peroxide test strips. The EPR spectra and CV results confirm the formation and spontaneous decay of ferryl intermediates upon the reaction of ferric state of HRP with H_2_O_2_. We successfully detected an adventitious amount of H_2_O_2_ in alcohols, such as ethanol, glycerol, 2-chloroethanol, and isopropanol. This demonstration suggests a simple, cost-effective, and accurate method for the detection of ultratrace amount of H_2_O_2_ impurity in alcohols using UV/Vis spectrophotometry, which enables the use of this method in biomedical, biological and chemical applications.

## Experimental section

### Materials

The salt-free, lyophilized powder of HRP isozyme C type VI-A (950–2000 units per mg solid), monobasic and dibasic potassium phosphate (≥98%), potassium ferricyanide (≥99.0%), and ABTS (≥98%) were purchased from Sigma Aldrich (St. Louis, MO). Hydrogen peroxide (30% w/w solution) was purchased from Anachemia (Que, Canada). Pure 200 proof ethanol was purchased from KOPTEC (PA, US) and glycerol was purchased from Mallinckrodt Chemicals (Wilkes-Barre, PA). Other alcohols including propan-2-ol, methanol, 2-mercaptoethan-1-ol, 2-chloroethan-1-ol, 2,2-dichloroethan-1-ol, 2,2,2-trichloroethan-1-ol, 2,2,2-trifluoroethan-1-ol, and ethylene glycol were purchased from Sigma Aldrich (St. Louis, MO).

### Detection of H_2_O_2_ through the analysis of the ferric state of HRP and its ferryl intermediate using UV/Vis spectrophotometry

Aqueous solutions of HRP at three different concentrations (1 μM, and 10 μM) were prepared in 0.1 M potassium phosphate buffer pH 6.0. Hydrogen peroxide (30% w/w) was initially diluted in ethanol to 0.01 M and then further diluted in ethanol to reaction concentrations through serial dilutions (0.001 μM, 0.01 μM, 0.1 μM, 1 μM, 10 μM, 100 μM, 1000 μM, and 10^4^ μM). The HRP solution (500 μL) was treated with 500 μL of each ethanol/H_2_O_2_ solution to form ferryl intermediates. The UV/Vis absorption spectra of the ferryl intermediates were collected using a UV-Vis Spectrophotometer UV-2600, Shimadzu Scientific Instruments/Marlborough, MA in the range of 200–800 nm. The spectra were investigated at the Soret and Q-band regions.

### Analysis of compound I and II formed from the ferric state of HRP upon the addition of H_2_O_2_ using UV/Vis spectrophotometry

UV/Vis absorption spectra of the native HRP solution (10 μM) and the HRP solution treated with aqueous solutions of H_2_O_2_ (100 μM) were obtained using UV/Vis spectrophotometry (UV-Vis Spectrophotometer UV-2600, Shimadzu Scientific Instruments/Marlborough, MA) in the scan range of 200–800 nm. The spectra were recorded immediately upon the addition of 500 μL of 100 μM H_2_O_2_ to 500 μL of 10 μM HRP solution in 0.1 M potassium phosphate buffer (pH 6.0).

### Analysis of the ferric state of HRP and its ferryl intermediate using cyclic voltammetry

The CV data was collected using a BASi EC Epsilon potentiostat. The reference electrode was a silver wire immersed in a saturated solution of KCl, the counter electrode consisted of a platinum wire coil with 10 cm length, and the working electrode was a glassy carbon electrode of 0.3 mm diameter. The working electrode was polished to a mirror-like finish on a pad with 0.3 μm alumina and deionized water, and then sonicated for 30 s in deionized water. The platinum counter electrode was burned with a butane flame for 30 s. The reference electrode solution was made fresh for every measurement and at the end of each experiment, a small amount of potassium ferricyanide was added as an internal reference. Anhydrous nitrogen gas was purged through the HRP solution for at least 10 minutes prior to analysis.

### Analysis of the ferric state of HRP and its ferryl intermediate using EPR

EPR spectra were recorded on a Bruker EMX (BRUKER, Billerica, MA) spectrometer at a frequency of 9.24 GHz under standard conditions in 4 mm ID quartz tubes. A liquid helium cryostat ESR-10 (Oxford Instruments Ltd, England) was used to stabilize the temperature at 12 K. The spectra were recorded with a modulation amplitude of 8G and microwave power of 625 μW.

### Quantification of H_2_O_2_ through the oxidation of ABTS

The H_2_O_2_ concentration was measured spectrophotometrically based on the oxidation of the substrate, 2,2′-azino-bis(3-ethylbenzothiazoline-6-sulfonic acid) diammonium salt (ABTS), in the presence of HRP using UV/Vis spectrophotometry (UV-Vis Spectrophotometer UV-2600, Shimadzu Scientific Instruments/Marlborough, MA). ABTS changes from colorless to blue-green in color upon oxidation, and the intensity of this color can be easily measured by UV/Vis spectrophotometry. 50 μL of ABTS stock solutions (2 mM, 20 mM) in 0.1 M potassium phosphate buffer (pH 6.0) were mixed with 50 μL of HRP solution (0.1 μM, 1 μM, and 10 μM) and 50 μL of each alcohol containing different concentrations of H_2_O_2_ (0.001 μM, 0.01 μM, 0.1 μM, 0.5 μM 1 μM, 10 μM, 50 μM, 100 μM, 500 μM, and 1000 μM). Different concentrations of H_2_O_2_ were prepared by serial dilution. By measuring the color intensity of ABTS at each concentration of H_2_O_2_, we optimized the best concentrations for measuring the range of H_2_O_2_ impurity. The ABTS absorbance at 420 nm (*λ*_max_ of the oxidized product of ABTS) was plotted *versus* H_2_O_2_ concentrations and used as calibration curves. Three main formulas were derived from the linear fits and were used to measure the H_2_O_2_ impurity in alcohols.

### Statistical analysis

Data were subjected to analysis of variance (ANOVA) using SPSS software package version 15.0 for Windows. All measurements were performed in triplicate. Mean comparisons were performed using the post hoc multiple comparison Duncan test to examine if differences were significant at *P* < 0.05.

## Conflicts of interest

There are no conflicts to declare.

## Supplementary Material

RA-011-D1RA00733E-s001

## References

[cit1] Rhee S. G., Kang S. W., Jeong W., Chang T.-S., Yang K.-S., Woo H. A. (2005). Curr. Opin. Cell Biol..

[cit2] Halliwell B., Gutteridge J. (1999). Free Radicals Biol. Med..

[cit3] Ohshima H., Tatemichi M., Sawa T. (2003). Arch. Biochem. Biophys..

[cit4] Shah A., Channon K. (2004). Heart.

[cit5] Barnham K. J., Masters C. L., Bush A. I. (2004). Nat. Rev. Drug Discovery.

[cit6] Usui Y., Sato K., Tanaka M. (2003). Angew. Chem., Int. Ed..

[cit7] Khorami H. A., Botero-Cadavid J. F., Wild P., Djilali N. (2014). Electrochim. Acta.

[cit8] Pozio A., Silva R., De Francesco M., Giorgi L. (2003). Electrochim. Acta.

[cit9] Kinumoto T., Inaba M., Nakayama Y., Ogata K., Umebayashi R., Tasaka A., Iriyama Y., Abe T., Ogumi Z. (2006). J. Power Sources.

[cit10] Salimi A., Hallaj R., Soltanian S., Mamkhezri H. (2007). Anal. Chim. Acta.

[cit11] Cohen G., Hochstein P. (1964). Biochemistry.

[cit12] Albers A. E., Okreglak V. S., Chang C. J. (2006). J. Am. Chem. Soc..

[cit13] Wang J. (2001). Electroanalysis.

[cit14] Quintino M. S., Winnischofer H., Araki K., Toma H. E., Angnes L. (2005). Analyst.

[cit15] Cai H., Liu X., Zou J., Xiao J., Yuan B., Li F., Cheng Q. (2018). Chemosphere.

[cit16] Yuan L., Lin W., Xie Y., Chen B., Zhu S. (2011). J. Am. Chem. Soc..

[cit17] King D. W., Cooper W. J., Rusak S. A., Peake B. M., Kiddle J. J., O'Sullivan D. W., Melamed M. L., Morgan C. R., Theberge S. M. (2007). Anal. Chem..

[cit18] Ju J., Chen W. (2015). Anal. Chem..

[cit19] Roberts J. G., Voinov M. A., Schmidt A. C., Smirnova T. I., Sombers L. A. (2016). J. Am. Chem. Soc..

[cit20] Yang X., Ma K. (2005). Anal. Biochem..

[cit21] Wen F., Dong Y., Feng L., Wang S., Zhang S., Zhang X. (2011). Anal. Chem..

[cit22] Dıaz A. N., Peinado M. R., Minguez M. T. (1998). Anal. Chim. Acta.

[cit23] Gosling J. P. (1990). Clin. Chem..

[cit24] Krainer F. W., Glieder A. (2015). Appl. Microbiol. Biotechnol..

[cit25] Fukuoka T., Tonami H., Maruichi N., Uyama H., Kobayashi S., Higashimura H. (2000). Macromolecules.

[cit26] Pappa H. S., Cass A. E. (1993). Eur. J. Biochem..

[cit27] Dequaire M., Limoges B., Moiroux J., Savéant J.-M. (2002). J. Am. Chem. Soc..

[cit28] Campomanes P., Rothlisberger U., Alfonso-Prieto M., Rovira C. (2015). J. Am. Chem. Soc..

[cit29] Erman J. E., Vitello L. B., Miller M. A., Kraut J. (1992). J. Am. Chem. Soc..

[cit30] Poulos T. L., Kraut J. (1980). J. Biol. Chem..

[cit31] Henriksen A., Smith A. T., Gajhede M. (1999). J. Biol. Chem..

[cit32] Casadei C. M., Gumiero A., Metcalfe C. L., Murphy E. J., Basran J., Teixeira S. C., Schrader T. E., Fielding A. J., Ostermann A., Blakeley M. P. (2014). Science.

[cit33] Karlin K. D. (2010). Nature.

[cit34] Sligar S. G. (2010). Science.

[cit35] Groves J. T. (2014). Nat. Chem..

[cit36] Patterson W. R., Poulos T. L., Goodin D. B. (1995). Biochemistry.

[cit37] Chreifi G., Baxter E. L., Doukov T., Cohen A. E., McPhillips S. E., Song J., Meharenna Y. T., Soltis S. M., Poulos T. L. (2016). Proc. Natl. Acad. Sci. U.S.A..

[cit38] Rodríguez-López J. N., Lowe D. J., Hernández-Ruiz J., Hiner A. N., García-Cánovas F., Thorneley R. N. (2001). J. Am. Chem. Soc..

[cit39] Dawson J. H. (1988). science.

[cit40] Hu L., Yuan Y., Zhang L., Zhao J., Majeed S., Xu G. (2013). Anal. Chim. Acta.

[cit41] Campos-Martin J. M., Blanco-Brieva G., Fierro J. L. (2006). Angew. Chem., Int. Ed..

[cit42] Ravanfar R., Lawrence P., Kriner K., Abbaspourrad A. (2019). J. Agric. Food Chem..

[cit43] Gazaryan I. G., Lagrimini L. M. (1996). Phytochemistry.

[cit44] Blumberg W., Peisach J., Wittenberg B. A., Wittenberg J. B. (1968). J. Biol. Chem..

[cit45] Dawson J. H., Sono M. (1987). Chem. Rev..

[cit46] Erman J. E., Yonetani T. (1975). Biochim. Biophys. Acta, Protein Struct..

[cit47] Wittenberg B. A., Kampa L., Wittenberg J. B., Blumberg W., Peisach J. (1968). J. Biol. Chem..

[cit48] Kwon H., Basran J., Casadei C. M., Fielding A. J., Schrader T. E., Ostermann A., Devos J. M., Aller P., Blakeley M. P., Moody P. C. (2016). Nat. Commun..

