# Supplementary material for: Monitoring the heme iron state in horseradish peroxidase to detect ultratrace amounts of hydrogen peroxide in alcohols
Source: RSC Adv. 2021 Mar 9;11(17):9901–10. doi: 10.1039/d1ra00733e (PMC8695524; doi:10.1039/d1ra00733e)
Supplement: RA-011-D1RA00733E-s001 [file RA-011-D1RA00733E-s001.pdf]

Electronic Supplementary Material (ESI) for RSC Advances.
This journal is © The Royal Society of Chemistry 2021

Monitoring the Heme Iron State in Horseradish Peroxidase to Detect Ultratrace Amount of Hydrogen Peroxide in Alcohols

*Raheleh Ravanfar, Alireza Abbaspourrad**

Department of Food Science, Cornell University, Ithaca, NY, USA

* Corresponding author: Alireza Abbaspourrad, e-mail: alireza@cornell.edu

Supplementary Figures

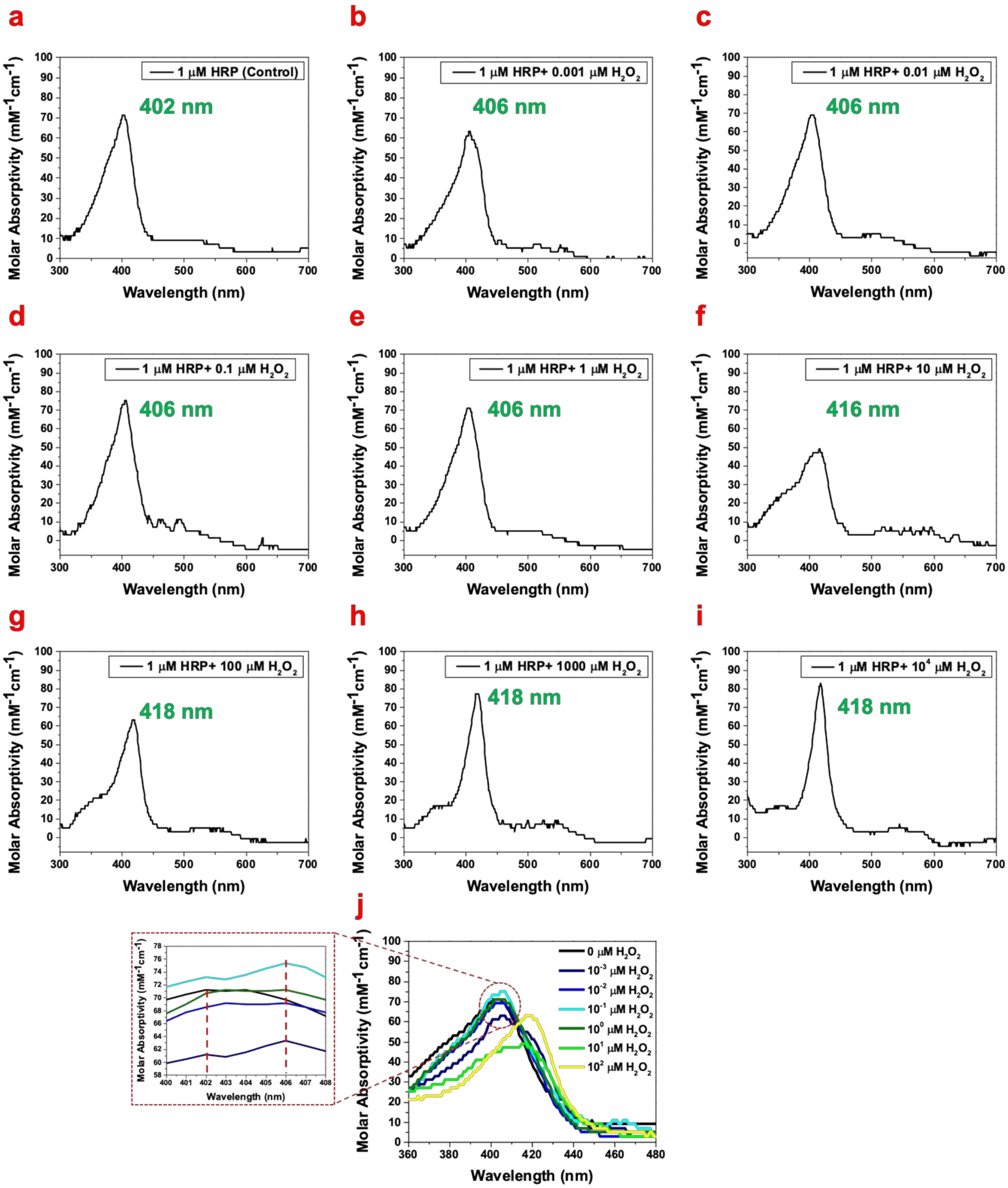

Figure S1. UV/Vis spectra of 1 μM Horseradish Peroxidase (500 μL) upon the addition of 500 μL of ethanol containing H_2O_2 at these concentrations a) 0 μM, b) 0.001 μM, c) 0.01 μM, d) 0.1 μM,

e) 1 μM, f) 10 μM, g) 100 μM, h) 1000 μM, and i) 10^4 μM. The wavelengths associated to Soret bands are shown in green. j) All the UV/Vis spectra of the 1 μM HRP solution upon the addition of H_2O_2 in ethanol at different concentrations are shown together.

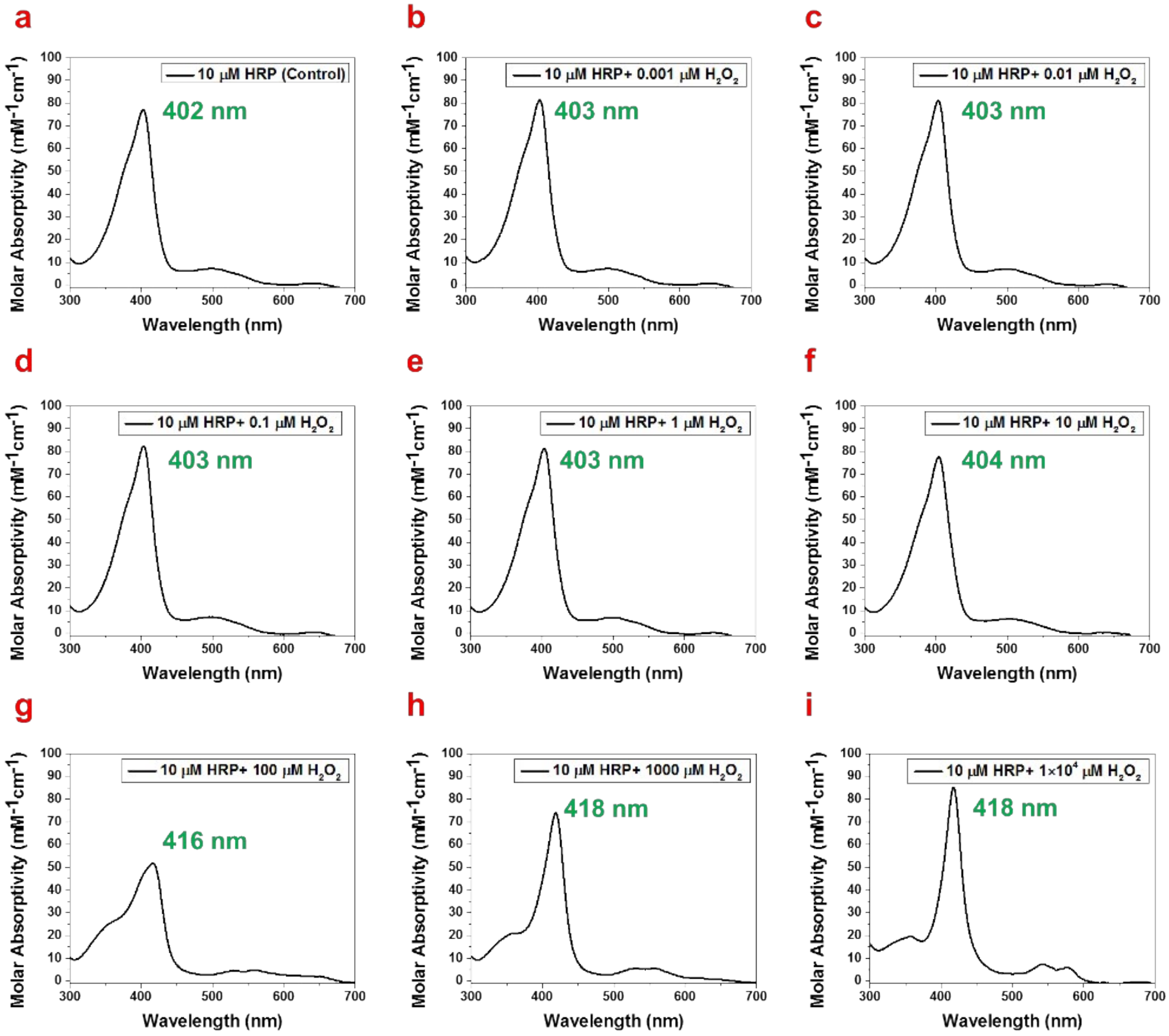

Figure S2. UV/Vis spectra of the 10 μM HRP solution in 0.1 M phosphate buffer (500 μL) upon the addition of 500 μL of ethanol containing H_2O_2 at these concentrations a) 0 μM, b) 0.001 μM, c) 0.01 μM, d) 0.1 μM, e) 1 μM, f) 10 μM, g) 100 μM, h) 1000 μM, and i) 10^4 μM. The wavelengths associated to Soret bands are shown in green. All HRP solutions were prepared in 0.1 M potassium phosphate buffer (pH= 6.0).

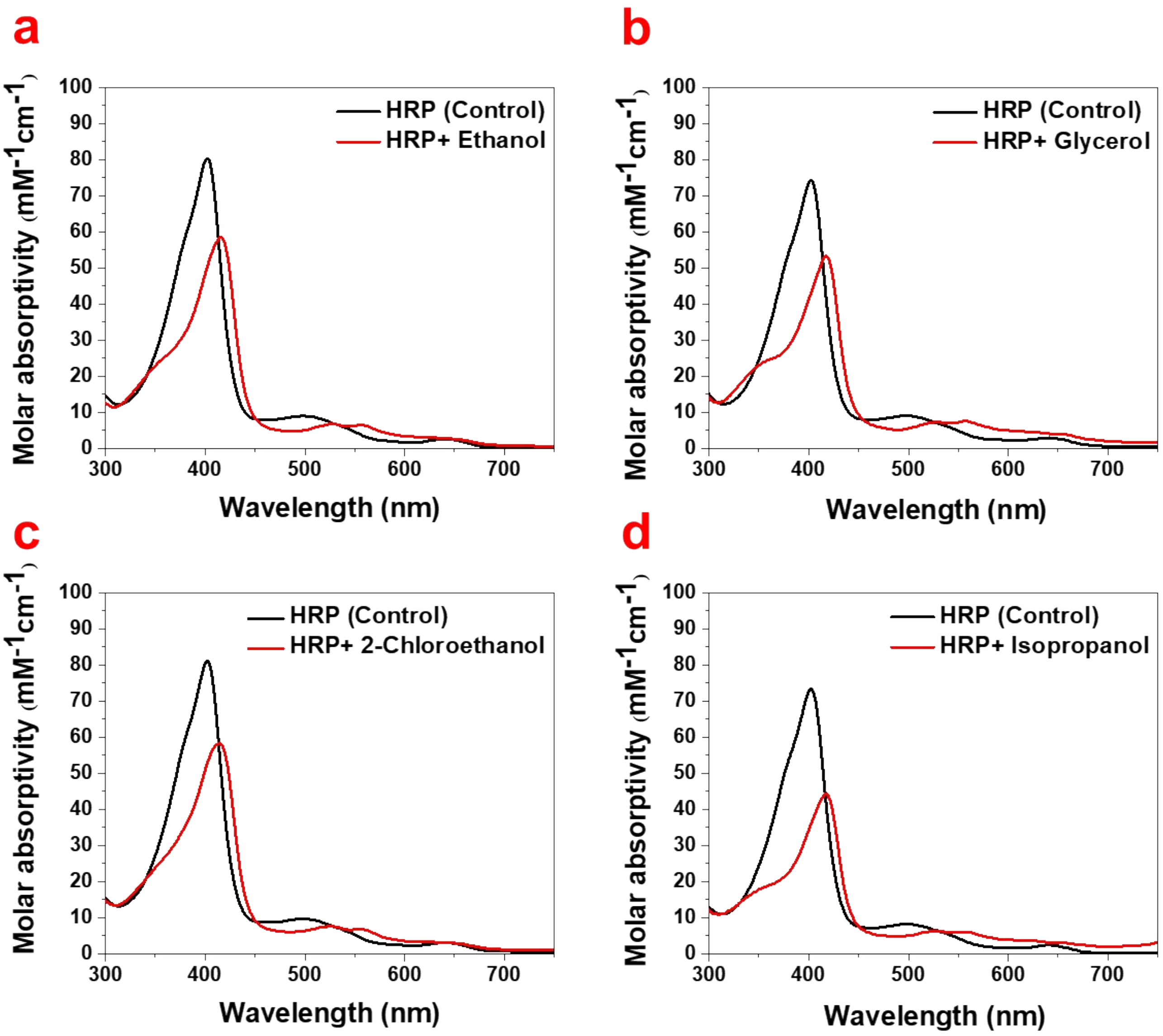

Figure S3. UV/visible absorption spectra of 500 μL HRP solution (10 μM) after treatment with 500 μL a) ethanol, b) glycerol, c) 2-chloroethanol, and d) isopropanol, containing unreported amounts of H_2O_2.

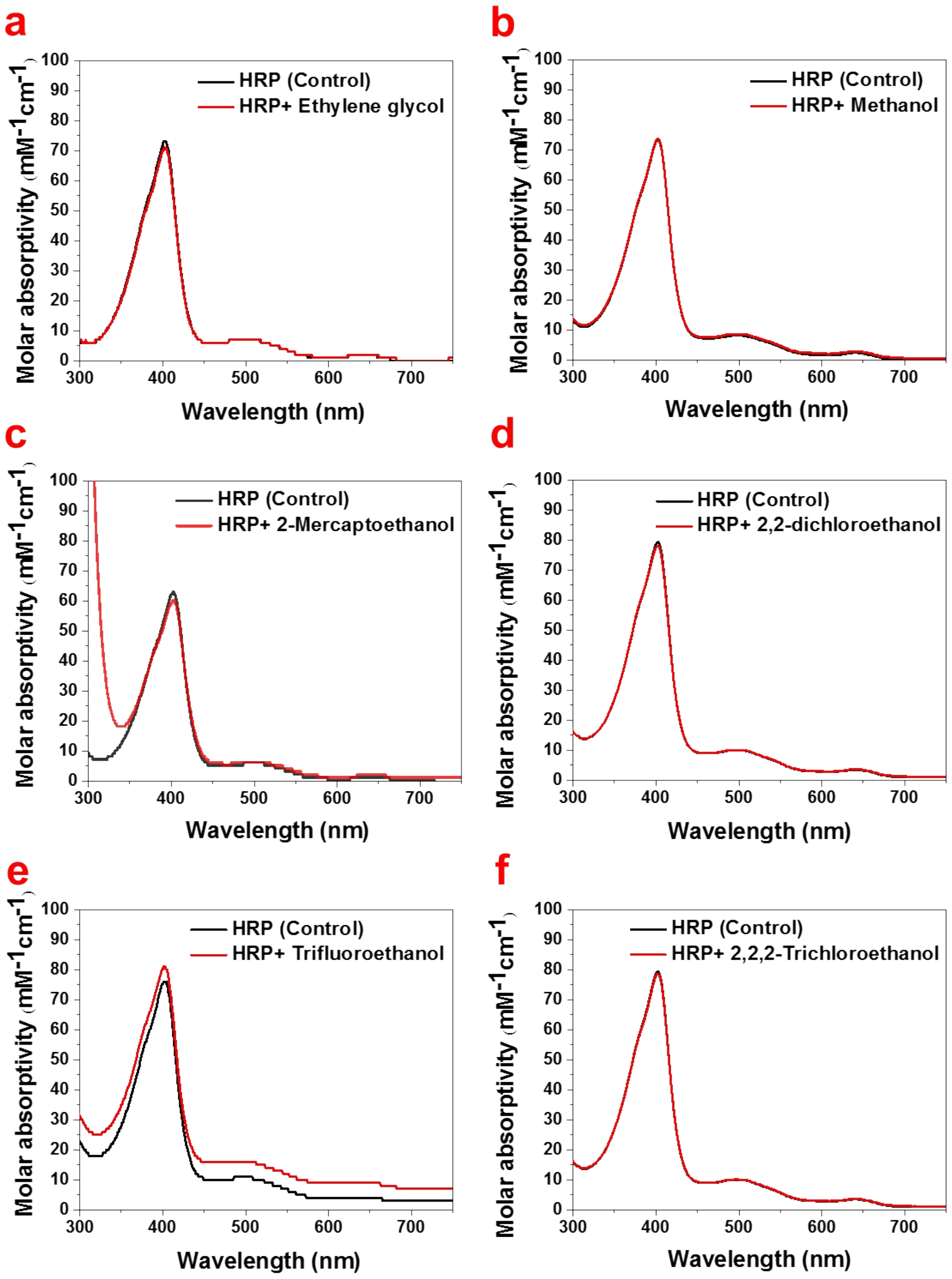
a
HRP (Control)
HRP+ Ethylene glycol
b
HRP (Control)
HRP+ Methanol
c
HRP (Control)
HRP+ 2-Mercaptoethanol
d
HRP (Control)
HRP+ 2,2-dichloroethanol
e
HRP (Control)
HRP+ Trifluoroethanol
f
HRP (Control)
HRP+ 2,2,2-Trichloroethanol
Molar absorptivity (mM^{-1}cm^{-1})
Wavelength (nm)
0
10
20
30
40
50
60
70
80
90
100
300
400
500
600
700

Figure S4. UV/visible absorption spectra of 500 μL HRP solution (10 μM) after treatment with 500 μL a) ethylene glycol, b) methanol, c) 2-mercaptoethanol, d) 2,2-dichloroethanol, e) trifluoroethanol, and f) 2,2,2,-trichloroethanol.

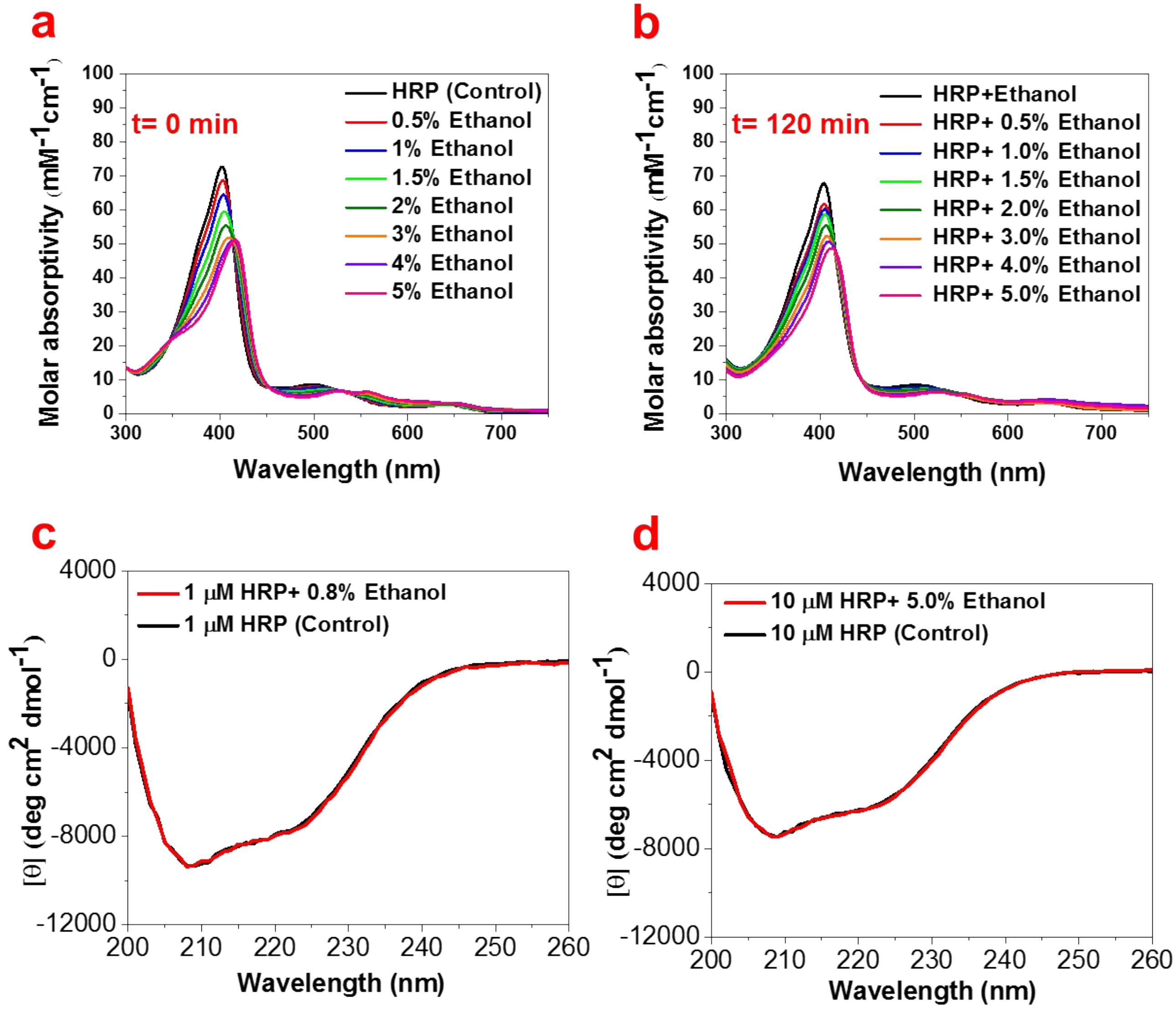

Figure S5. The red shifts upon a) the first addition of ethanol containing 100 μM H_2O_2 and b) the second addition of ethanol containing 100 μM H_2O_2 after the decay of the ferryl intermediate formed from the first addition of ethanol. c) Circular dichroism spectroscopy for 500 μL of 1 μM

HRP solution in potassium phosphate buffer (0.1 M, pH= 6.0) before and after treatment with 500 μL ethanol containing 80 μM H_2O_2. d) Circular dichroism spectroscopy of 500 μL of 10 μM HRP solution in potassium phosphate buffer (0.1 M, pH= 6.0) before and after treatment with 500 μL ethanol containing 80 μM H_2O_2.

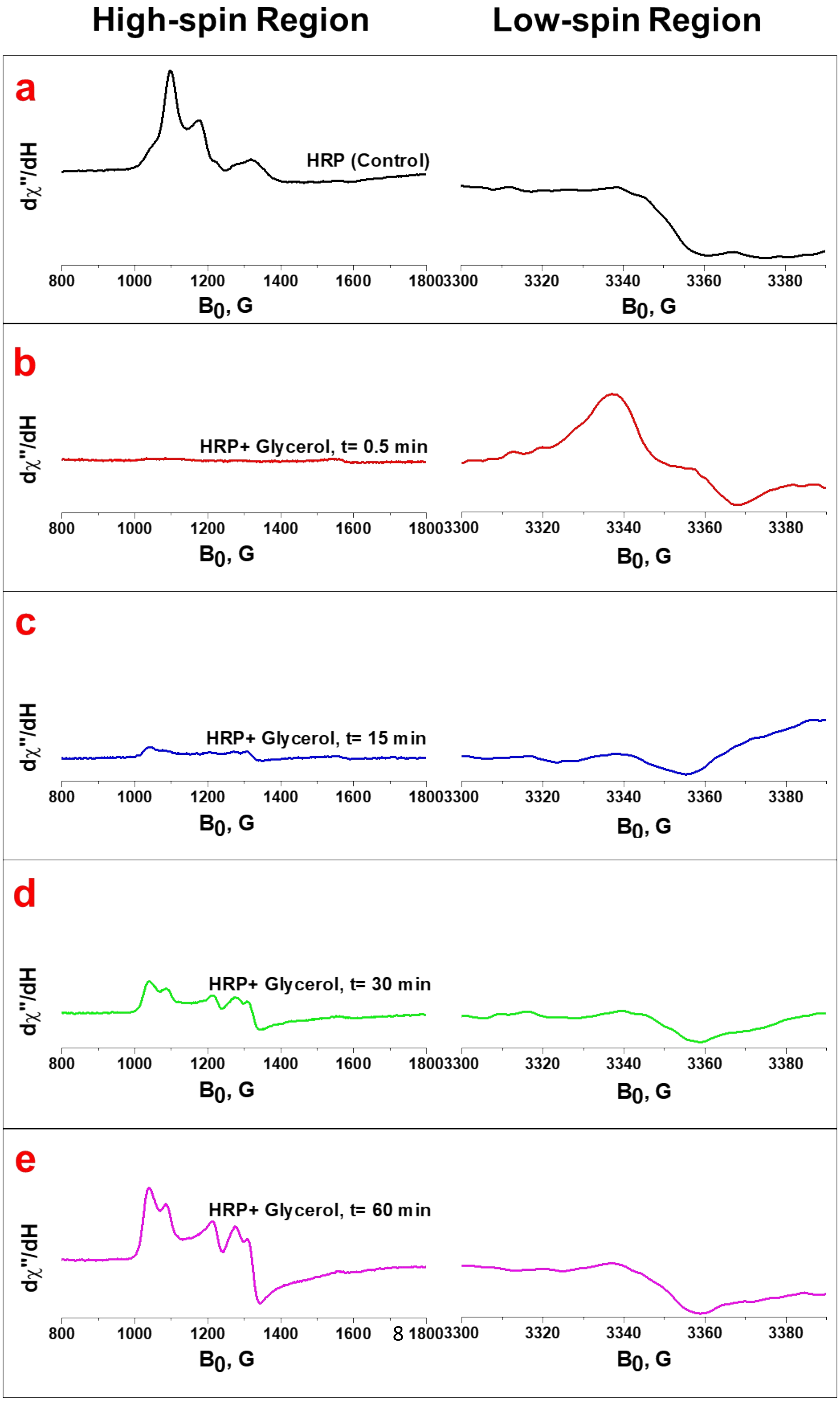

High-spin Region
Low-spin Region
a
HRP (Control)
b
HRP+ Glycerol, t= 0.5 min
c
HRP+ Glycerol, t= 15 min
d
HRP+ Glycerol, t= 30 min
e
HRP+ Glycerol, t= 60 min
dχ''/dH
B0, G
800 1000 1200 1400 1600 1800
3300 3320 3340 3360 3380

Figure S6. a) EPR spectra of the native HRP (control) at both high-spin and low-spin regions. b, c, d, e) EPR spectra of the 100 μL HRP solution upon the addition of 100 μL glycerol containing H_2O_2 impurity at 0.5 min, 15 min, 30 min, and 60 min, respectively. Spectra were collected at 12 K and 625 μW.

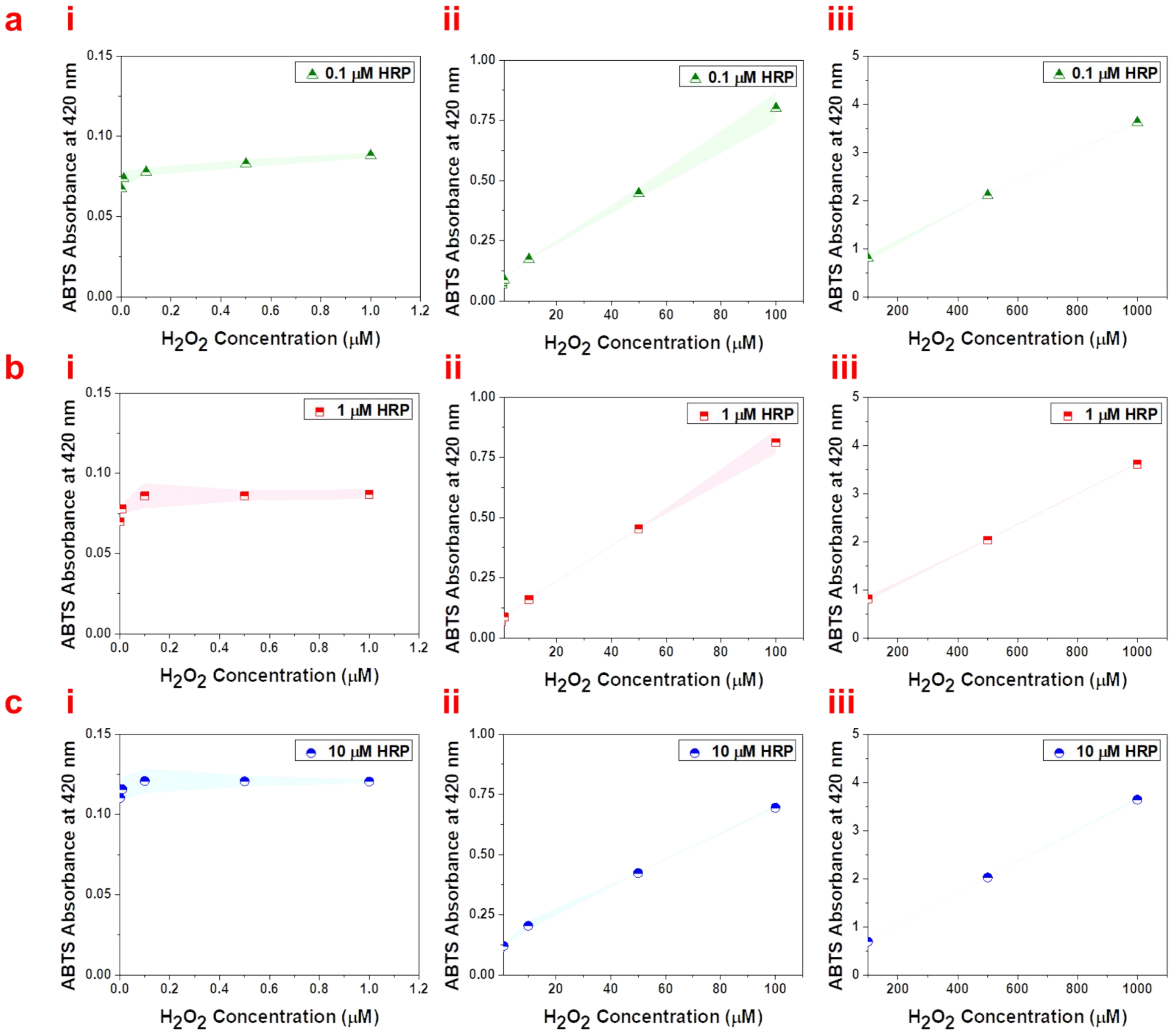

Figure S7. UV/Vis absorbance of 2 mM ABTS at 420 nm at different ranges of H_2O_2 impurity of ethanol (i: 0 μM – 1 μM, ii: 1 μM – 100 μM, and iii: 100 μM – 1000 μM) using HRP solution concentrations of a) 0.1 μM, b) 1 μM, and c) 10 μM. 50 μL of ABTS in 0.1 M potassium phosphate buffer (pH 6.0) was mixed with 50 μL of HRP solution and 50 μL of ethanol containing different concentrations of H_2O_2. The absorbance at 420 nm (λ_{max} of the oxidized product of ABTS) was recorded versus H_2O_2 concentrations at 25 ºC 30 min after mixing, which was linearly increased.

Table S1. The formulas to measure the impurity of H_2O_2 in alcohols based on the absorbance range of ABTS. Briefly, 50 μL of ABTS (20 mM) in 0.1 M potassium phosphate buffer (pH 6.0) was mixed with 50 μL of HRP solution (0.1 μM) and 50 μL of ethanol containing H_2O_2. The absorbance at 420 nm (λ_{max} of the oxidized product of ABTS) was recorded versus H_2O_2 concentrations at 25 ºC, 30 min after mixing, which was linearly increased. Three main formulas were derived from the linear fits and were used to measure the H_2O_2 impurity in alcohols.

Formula No.	Formula	R^2	Range of ABTS absorbance (a.u)	Range of H_2O_2 impurity (μM)
1	$y = 0.081x + 0.13$	0.97	0-0.3	0 - 1
2	$y = 0.005x + 0.20$	0.99	0.3-1.0	1 - 100
3	$y = 0.003x + 0.37$	0.99	1.0-4.0	100 - 1000

Table S2. Quantification of H_2O_2 impurity in common primary and secondary alcohols. 50 μL of ABTS (20 mM) in 0.1 M potassium phosphate buffer (pH 6.0) was mixed with 50 μL of HRP solution (0.1 μM) and 50 μL of alcohols containing impurity of H_2O_2. The absorbance at 420 nm (λ_{max} of the oxidized product of ABTS) was recorded versus H_2O_2 concentrations at 25 ºC 30 min after mixing at 1 cm path length, which was linearly increased. The mean and standard deviation for three absorbance measurements of three samples prepared similarly have been shown in the second column from left.

Alcohol	ABTS radical cation absorbance (a.u.), 1 cm path length	Applied Formula No.	H_2O_2 impurity (μM)
Ethanol	0.600± 0.013	2	79.9
Glycerol	0.279± 0.003	1	1.8
Benzyl alcohol	3.479± 0.003	3	1036.4
2-Chloroethanol	1.844± 0.026	3	491.4
1-Butanol	0.187± 0.005	1	0.7
1-Hexanol	0.304± 0.004	1	2.1
1-propanol	0.191± 0.002	1	0.8
Isopropanol	0.136± 0.008	1	0.1
